# Dissimilarity in the Folding of Human Cytosolic Creatine Kinase Isoenzymes

**DOI:** 10.1371/journal.pone.0024681

**Published:** 2011-09-09

**Authors:** Yin Wang, Sha Wang, Yan-Song Gao, Zhe Chen, Hai-Meng Zhou, Yong-Bin Yan

**Affiliations:** 1 State Key Laboratory of Biomembrane and Membrane Biotechnology, School of Life Sciences, Tsinghua University, Beijing, China; 2 Protein Science Laboratory of the Ministry of Education, School of Life Sciences, Tsinghua University, Beijing, China; 3 Zhejiang Provincial Key Laboratory of Applied Enzymology, Yangtze Delta Region Institute of Tsinghua University, Zhejiang, China; University of South Florida College of Medicine, United States of America

## Abstract

Creatine kinase (CK, EC 2.7.3.2) plays a key role in the energy homeostasis of excitable cells. The cytosolic human CK isoenzymes exist as homodimers (HMCK and HBCK) or a heterodimer (MBCK) formed by the muscle CK subunit (M) and/or brain CK subunit (B) with highly conserved three-dimensional structures composed of a small N-terminal domain (NTD) and a large C-terminal domain (CTD). The isoforms of CK provide a novel system to investigate the sequence/structural determinants of multimeric/multidomain protein folding. In this research, the role of NTD and CTD as well as the domain interactions in CK folding was investigated by comparing the equilibrium and kinetic folding parameters of HMCK, HBCK, MBCK and two domain-swapped chimeric forms (BnMc and MnBc). Spectroscopic results indicated that the five proteins had distinct structural features depending on the domain organizations. MBCK BnMc had the smallest CD signals and the lowest stability against guanidine chloride-induced denaturation. During the biphasic kinetic refolding, three proteins (HMCK, BnMc and MnBc), which contained either the NTD or CTD of the M subunit and similar microenvironments of the Trp fluorophores, refolded about 10-fold faster than HBCK for both the fast and slow phase. The fast folding of these three proteins led to an accumulation of the aggregation-prone intermediate and slowed down the reactivation rate thereby during the kinetic refolding. Our results suggested that the intra- and inter-subunit domain interactions modified the behavior of kinetic refolding. The alternation of domain interactions based on isoenzymes also provides a valuable strategy to improve the properties of multidomain enzymes in biotechnology.

## Introduction

**Figure 1:**
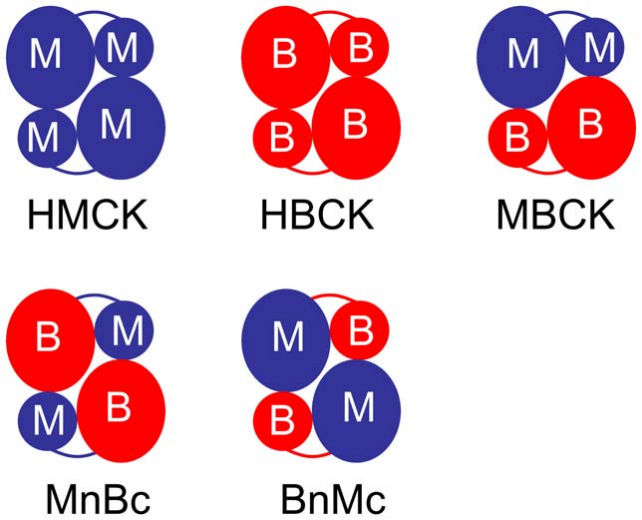
Domain and subunit organizations of the homodimeric CKs (HMCK and HBCK), the heterodimeric form (MBCK) and the two domain-swapped chimeric forms (BnMc and MnBc).

Creatine kinase (CK, adenosine-5′-triphosphokinase: creatine phosphotransferase; EC 2.7.3.2) catalyzes the reversible transfer of the N-phosphoryl group from phosphocreatine (PCr) to ADP to generate ATP and creatine (Cr) thereby. CK plays a key role in the energy homeostasis of excitable cells by connecting the sites of ATP generation and consumption and buffering the intracellular ATP/ADP ratio [1], and thus participates in many vital physiological processes [2]–[7]. In vertebrates, two cytosolic and two mitochondrial CK isoenzymes have been identified with distinct tissue and subcellular distributions [8]–[11]. Among them, the cytosolic CK isoforms can exist as homodimers (MMCK and BBCK) or a heterodimer (MBCK) formed by the muscle CK subunit (M) and/or brain CK subunit (B) with similar molecular masses (Figure 1). The muscle type CK mainly distributes in muscles and heart, while the brain type CK is highly expressed in many tissues such as brain, nervous system, retina and heart [8], [11]. The two cytosolic isoforms are almost superimposed in their backbone structures, and each subunit of both proteins composes a small N-terminal domain (NTD) and a large C-terminal domain (CTD). The discrepancy between the structures of MMCK and BBCK is mainly located at the N-terminus and several loops in the CTD [12]–[15].

Despite of the highly conserved sequence and three-dimensional structure [16], the two cytosolic CK isoenzymes are quite distinct in their biological features. Particularly, MMCK specifically binds to the myofibrils [2], [17], [18], which is not shared by BBCK. Moreover, although MMCK and BBCK mainly exists in the reduced form with no intramolecular disulfide bond, both of them can exist in an oxidized form by the introduction of an intra-subunit disulfide bond, but the position and role of the disulfide bond is quite different [3], [19]. The distinct tissue distribution and physiological functions may endow the two cytosolic isoenzymes different thermal stability to adapt the dissimilar microenvironments and functional requirements during evolution [20]–[22].

Rabbit MMCK (RMCK) has been taken as a model in protein folding studies for several decades. Previous studies have shown that the equilibrium folding of RMCK is dominated by a three-state process with the appearance of a monomeric molten globular (MG) intermediate [23]–[27]: 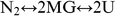
(Scheme 1)where N_2_ is the native state and U is the unfolded state. The kinetic folding of RMCK is a multi-step process involving several kinetic intermediates [24], [28]–[34]. In summary, the kinetic refolding of RMCK initiated by manual mixing is dominated by a biphasic process. The fast phase involves the formation of a partially active dimeric intermediate (N_2_′), while the slow phase produces a native-like enzyme (N_2_^*^). The appearance of an inactive dimeric intermediate (U_2_) is a very fast process during refolding, and U_2_ is prone to aggregate and can be trapped by molecular chaperones or osmolytes. A simplified model of RMCK refolding from denaturants is shown in Scheme 2 (modified from [29], [33]).
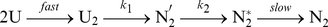
(Scheme 2)


The highly conserved overall fold suggests that the cytosolic CKs may follow a similar folding pathway, while the differences in sequence and structure may affect the thermodynamic and kinetic parameters of folding. Thus the isoforms of CK provide a good system to investigate the sequence/structural determinants of multimeric/multidomain protein folding. In this research, the role of NTD and CTD as well as the domain interactions in CK folding was investigated by comparing the equilibrium and kinetic folding of human MMCK (HMCK), human BBCK (HBCK), the hybrid form (MBCK) and two domain-swapped chimeric forms (BnMc and MnBc).

## Materials and Methods

### Materials

All restriction enzymes used in this study were purchased from New England Biolabs or TaKaRa. Ultrapure guanidine hydrochloride (GdnHCl), Tris, sodium dodecylsulfate (SDS), isopropyl-1-thio-β-d-galactopyranoside (IPTG), 1-anilinonaphtalene-8-sulfonate (ANS) and ATP were Sigma products. All other chemicals were local products of analytical grade or higher.

### Protein expression and purification

The genes of the wild-type (WT) human CKs were obtained as described elsewhere [21]. The chimeras BnMc and MnBc (Figure 1) were obtained by PCR method using the following primers: MnBc-For, 5′-GAAAACCTCAAGGGCGGCGACGACCTGGAC-3′; MnBc-Rev, 5′-GTCGCCGCCCTTGAGGTTTTCATGGTTGAG-3′; BnMc-For, 5′-GACAACCTGCAGGGTGGAGACGACCTGGAC-3′; and BnMc-Rev, 5′-GTCGTCTCCACCCTGCAGGTTGTCGGGGTTG-3′. All enzymes were overexpressed in *Escherichia coli* BL21 [DE3]-pLysS (Stratagene, Germany) and purified as described previously [21], [35]. In brief, the *E. coli* cells were cultured at 37°C for 3 h to reach an OD_600_ of about 0.8, and then the overexpression of the proteins were induced by the addition of 0.4 mM IPTG. After incubation at 16°C for 24 h, the cells were harvested and resuspended in 5 mM Tris-HCl, pH 9.0 (hMMCK) or pH 8.0 (hBBCK, hMBCK, BnMc and MnBc). The supernatant of the cell lysate was loaded onto a DEAE Sepharose Fast Flow anion-exchange column (Pharmacia) after centrifugation at 20 000 g for 30 min. The enzymes were eluted using the same buffer with a linear NaCl gradient from 0 to 0.6 M, and were then concentrated and loaded onto a Sephacryl S300 HR column (Pharmacia). The final products were dissolved in 20 mM Tris-HCl, pH 8.0, and the purity of the final products was above 98%. The protein concentration was determined according to the Bradford method by using bovine serum albumin as a standard [36]. All protein samples were freshly prepared to avoid the loss of activity after storage.

### Activity assay

CK activity was determined according to the pH colorimetry method in the forward direction (phosphocreatine formation) using the same condition as those described previously [37]. The enzymatic activity was monitored by the absorption at 597 nm with an Ultraspec 4300 pro UV-visible spectrophotometer (Amersham Pharmacia Biotech). All activity measurements were performed at 25°C, and were conducted for at least three repetitions.

### Spectroscopic experiments

Details regarding the spectroscopic characterization of the recombinant proteins were the same as those described previously [35]. In brief, all spectroscopic experiments were performed at 25°C using a protein concentration of 0.2 mg/ml. Far-UV circular dichroism (CD) were measured on a Jasco-715 spectrophotometer using 0.1-cm-pathlength cells with a resolution of 0.5 nm. The fluorescence emission spectra were collected on a Hitachi F-2500 spectrofluorimeter using a split width of 5 nm and a resolution of 0.5 nm. The intrinsic Trp fluorescence was exited at 295 nm, while the ANS extrinsic fluorescence was excited at 380 nm. All the fluorescence experiments were conducted at least three times.

### Equilibrium unfolding and refolding by GdnHCl

The equilibrium protein unfolding were carried out by incubating 0.2 mg/ml proteins in 20 mM Tris-HCl buffer (pH 8.0) containing various concentrations of GdnHCl at 25°C overnight. As for the equilibrium refolding experiments, the fully-unfolded proteins were prepared by denaturing 24 mg/ml proteins in 6 M GdnHCl for 1 h. The fully-denatured states of the proteins were characterized by spectroscopic methods, which revealed that all proteins were in the states with the lack of any regular secondary structures and the fully-exposed Trp residues (data not shown). The refolding was conducted by a manual dilution (1∶120) of the denatured proteins in 20 mM Tris-HCl buffer (pH 8.0) containing various concentration of GdnHCl, and incubated at 25°C overnight. The far-UV CD, intrinsic and ANS spectra and turbidity at 400 nm for each sample were recorded to monitor the structural changes during equilibrium unfolding and refolding.

### Kinetic refolding and reactivation of the GdnHCl-denatured proteins

The fully-unfolded proteins were prepared by denaturing 24 mg/ml proteins in 20 mM Tris-HCl buffer (pH 8.0) containing 6 M GdnHCl for 1 h. The refolding was initiated by a manual dilution (1∶200) of the denatured proteins in 20 mM Tris-HCl buffer (pH 8.0). No aggregates were observed under the above refolding conditions. The dead time of the manual mixing in kinetic studies was about 2 s. The refolding kinetics was measured according to previous methods [35] by monitoring the intrinsic fluorescence emission at 350 nm (excited at 295 nm) versus time, and the data were recorded every 2 s. The reactivation was monitored by measuring the recovered activity of the samples quenched at suitable time intervals. The activity data were normalized by taking the activity of the native enzymes as 100%. The time-course aggregation during refolding was monitored by the turbidity at 400 nm after a 1∶120 manual fast dilution of the denatured proteins in 20 mM Tris-HCl buffer, and the data were recorded every 2 s.

## Results

The enzymatic and structural features of the five enzymes are summarized in Table 1. A comparison of the enzymatic parameters indicated that all the enzymes possessed similar catalytic properties. Consistent with previous observations [21], [38], the catalytic efficiency of HBCK is higher than HMCK with stronger substrate binding affinity as reflected by a relative smaller *K*_m_ values for ATP and Cr. The catalytic efficiency is mainly determined by the nature of CTD since the activity of the chimera is similar to the one shared the same CTD, but not the one with the same NTD. The exchange of either NTD or CTD of HMCK by the corresponding domains in HBCK resulted in a decrease in the *K*_m_ values of ATP and Cr, which is close to those of HBCK. The substitution of domains led to a decrease in the *K*_m_-Cr value is consistent with the fact that the binding of Cr is stabilized by residues located both at NTD and CTD [12]. However, the ATP or ADP binding site is located at CTD, and the decrease in the *K*_m_-ATP values of the chimeras when compared to that of HMCK suggested that the domain interactions contributed to the stabilization of the conformation of the active site.

**Table 1:** Enzymatic parameters and biophysical properties of CKs.

	HBCK	HMCK	MBCK	BnMc	MnBc
*K*_m_-ATP (mM)	0.095±0.007	0.21±0.02	0.0830±0.007	0.107±0.008	0.120±0.009
*K*_m_-Cr (mM)	7.0±0.6	18±4	10±2	6.2±0.7	8.2±0.6
Relative Activity (%)	133	100	60	96	149
*E*_max_ (nm)	335	331	335	332	332
*I*_320_/*I*_365_	1.59	2.08	1.61	2.05	1.94
ANS Intensity (%)	217	100	168	102	218

**Figure 2:**
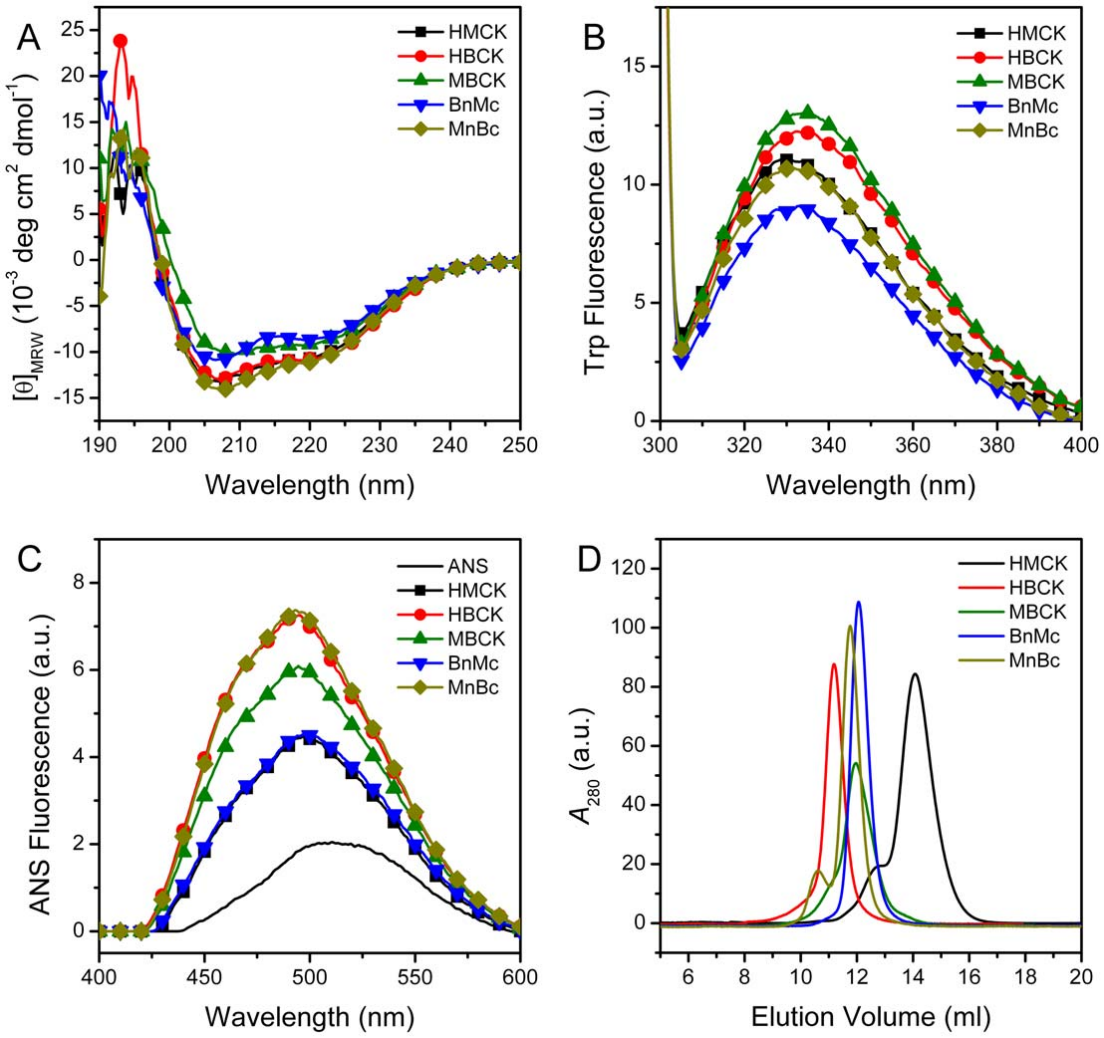
Biophysical characterization of the proteins. (A) Far-UV CD. (B) Intrinsic Trp fluorescence. (C) Extrinsic ANS fluorescence. (D) SEC analysis. The proteins were dissolved in 20 mM Tris-HCl buffer, pH 8.0. The protein concentration was 1 mg/ml for SEC analysis, and 0.2 mg/ml for spectroscopic experiments. The far-UV CD and Trp fluorescence spectra were obtained by subtracting the spectra of the buffer.

All enzymes contain similar regular secondary structure contents as revealed by the almost superimposed far-UV CD spectra, while MBCK and BnMc might be relatively less structured as indicated by the smaller absolute values of ellipticity (Figure 2A). All of the four Trp residues are located in CTD of CK, and previous fitting results indicated that RMCK have only one Class I fluorophore centered at 330 nm [22]. Two parameters were used to reflect the properties of the Trp fluorescence spectra: the emission maximum of the intrinsic Trp fluorescence (*E*_max_) and the *I*_320_/*I*_365_ value, also called Parameter *A*, which is a sensitive monitor of the shape and position of the Trp fluorescence [39]. The *E*_max_ of HMCK was at 331 nm, suggesting that the microenvironment of Trp residues in HMCK was the same as that in RMCK. The *E*_max_ of HBCK was about 4 nm red-shift when compared with HMCK (Figure 2B), and the fitting using the discrete Trp fluorophore model [40] indicated that about 80% of the fluorescence was contributed by Class II fluorophore centered at 340 nm, while the remainder was from Class I (data not shown). This suggested that the Trp residues in HBCK were more accessible to solvent although the two proteins are highly conserved in three-dimensional structure. The properties of the two chimeras were more close to that of HMCK as shown by the similar *E*_max_ and *I*_320_/*I*_365_ values listed in Table 1. The fact that the substitution of either NTD or CTD could blue-shift the Trp fluorescence of HBCK suggested that the alterations in domain interactions could modify the microenvironment of the Trp residues in CTD. However, MBCK had the same *E*_max_ value as HBCK. A comparison of the domain organization (Figure 1) and Trp fluorescence properties (Figure 2B) suggested that the Trp fluorophore properties were independent of the nature of CTD or subunit interactions, but mainly depended on the nature of the intra-subunit domain interactions. ANS extrinsic fluorescence indicated that HMCK and BnMc had the smallest hydrophobic exposure, while HBCK and MnBc had the largest (Figure 2C). This suggested that the major difference in the hydrophobic exposure was in CTD of cytosolic CKs. The discrepancy between the intrinsic and extrinsic fluorescence results suggested that the exposure of hydrophobic pockets did not correlate well with the solvent accessibility of the Trp fluorophores, which implied that the binding site(s) of the ANS molecule in CTD of B type CKs was not adjacent to the Trp residues. SEC analysis of the quaternary structure of the five proteins (Figure 2D) indicated that HMCK had the most compact structure as revealed by the largest elution volume, HBCK was the loosest one, while MBCK and the two chimeras was between HMCK and HBCK. It is worth noting that HMCK and MnBc were prone to form tetrameric forms after storage at a relative larger concentration, i.e. 1 mg/ml, while the other three proteins were not. The freshly-prepared proteins at low concentrations were dominated by the dimeric form, as shown previously [21].

**Figure 3:**
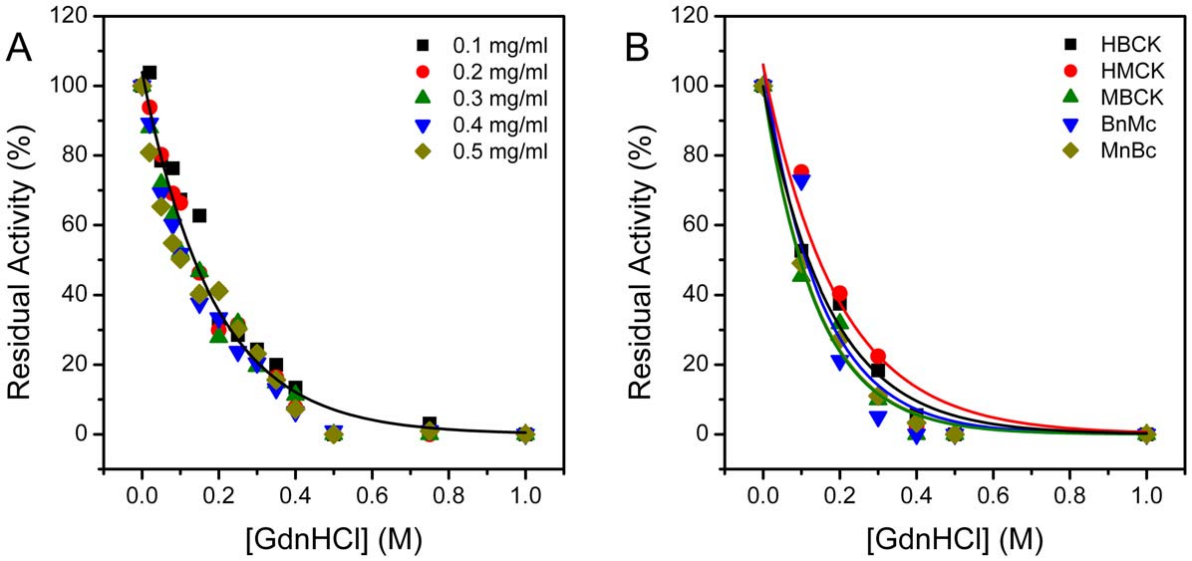
Inactivation of CKs by GdnHCl. (A) Concentration-dependence of HBCK inactivation by GdnHCl. The other four enzymes had similar inactivation curves independent on enzyme concentration (data not shown). (B) Inactivation of the five enzymes by GdnHCl.

**Figure 4:**
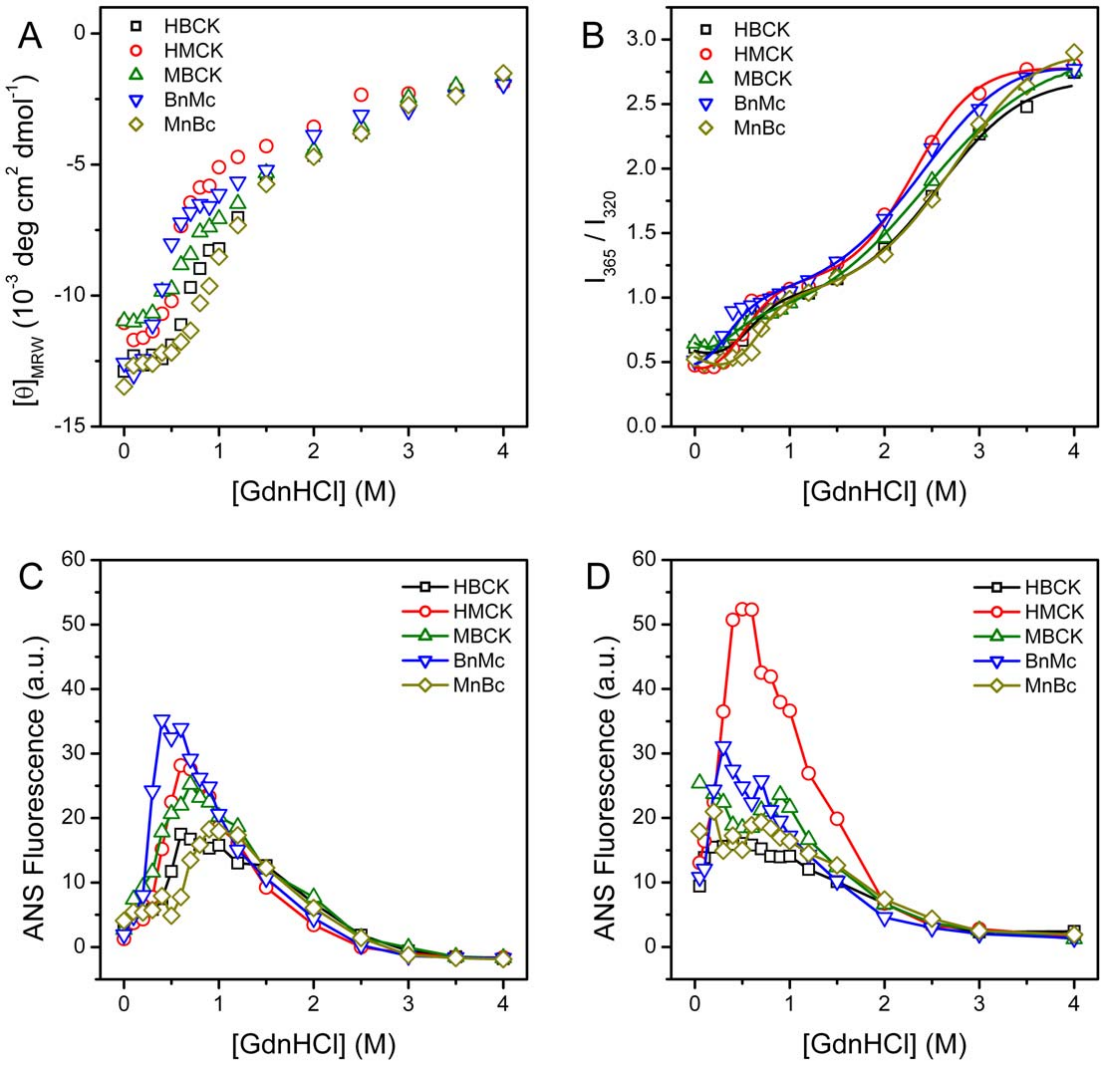
Equilibrium folding of CKs. (A) Mean residue molar ellipticity at 222 nm during unfolding. (B) Parameter A (*I*_365_/*I*_320_) of intrinsic fluorescence during unfolding. The data were fitted by the three-state model shown in Scheme 1, and the thermodynamic parameters were listed in Table 2. The fitted curves are presented as solid lines. (C) ANS fluorescence during unfolding. (D) ANS fluorescence during refolding. For clarity, the transition curves of refolding monitored by CD and Trp fluorescence are not shown. Protein unfolding were carried out by incubating 0.2 mg/ml proteins in 20 mM Tris-HCl buffer (pH 8.0) containing various concentrations of GdnHCl at 25°C overnight. The fully-unfolded proteins were prepared by denaturing 24 mg/ml proteins in 6 M GdnHCl for 1 h, and the refolding was conducted by a manual dilution (1∶120) of the denatured proteins in 20 mM Tris-HCl buffer (pH 8.0) containing various concentration of GdnHCl and incubated at 25°C overnight.

Similar to RMCK [35], [37], all the five enzymes used in this research were easily inactivated by GdnHCl, and the inactivation was independent on the enzyme concentration (Figure 3). Only slight difference was observed in the midpoints of inactivation for the five CKs, suggesting that the dissimilar domain organizations did not significantly affect the stability of the active site located in the cleft between NTD and CTD. The equilibrium folding of the five proteins was dominated by a similar three-state folding pathway (Scheme 1) as revealed by the transition curves monitored by CD, intrinsic and extrinsic fluorescence (Figure 4). A previous study has shown that in addition to the MG state, one additional intermediate (PMG) may appeared at a relatively higher GdnHCl concentration during the unfolding of RMCK [23]. The slight discrepancy between the transition curves from CD and fluorescence in Figure 4 also suggested that there might have more than one intermediate state for the human CKs. This could also be evidenced by the shape of the ANS fluorescence, which was more likely to be the sum of two populations. A comparison of the ANS fluorescence suggested that the alternations of the domain interactions might stabilize either MG or PMG. Particularly, the difference between the ANS fluorescence suggested that CTD of HMCK might stabilize MG appeared at a relatively lower GdnHCl concentration, while CTD of HBCK might stabilize PMG at a relatively higher GdnHCl concentration. Unfortunately, the existence of the PMG could not be extracted from the CD or fluorescence data. The phase diagram of Trp fluorescence, a sensitive tool to find the equilibrium folding intermediate [41], contained two linear parts (data not shown, similar to that in the previous study [42]), which implied that only one intermediate could be identified by Trp fluorescence data. This observation may be caused by the limitation of the Trp fluorescence probe or the undistinguishable properties of the two intermediates in Trp fluorescence. In this case, the three-state model in Scheme 1 was used for fitting.

**Table 2:** Thermodynamic parameters of CK folding fitted by a three-state model.

	HBCK	HMCK	MBCK	BnMc	MnBc
Δ*G*_NI_ (kJ mol^−1^)	39±3	36±2	35±2	34±7	42±2
*m*_NI_ (kJ mol^−1^ M^−1^)	17±1	15±1	18±2	16±3	19±2
Δ*G*_IU_ (kJ mol^−1^)	14±4	16.9±0.1	9.6±0.1	11.8±0.1	13.8±0.2
*m*_IU_ (kJ mol^−1^ M^−1^)	5.2±0.1	7.3±0.1	4.0±0.1	4.7±0.2	4.7±0.1
Δ*G*_total_ (kJ mol^−1^)	67	70	55	58	70

To minimize the possible errors introduced by variations in protein concentration of the samples, the fluorescence data were used to extract the thermodynamic parameters by nonlinear least square fitting using Scheme 1. As shown in Table 2, HBCK, HMCK and MnBc had similar stabilities, while MBCK and BnMc were less stable with lower Δ*G* values for both transitions. Since the N to MG transition involved the disruption the subunit interface and the alternation of the domain interactions [24], [35], [43], it seemed that among the five proteins, the interaction between NTD of HBCK and CTD of HMCK was the weakest, while the other patterns had comparable stabilities. Our results also suggested that the difference in the stabilities of the domains were minor for different isoenzymes, and the domain interactions played a crucial role in CK folding.

Consistent with previous results of RMCK [28], [30], [33], the folding of all five proteins were partially reversible as revealed by the inconsistency of the ANS fluorescence during folding and refolding. A comparison of the ANS fluorescence during CK folding and unfolding indicated that among the five proteins, the maximum ANS fluorescence of HMCK during refolding was significantly higher than that during unfolding. This suggested that the refolding of HMCK was prone to accumulate the MG state with high hydrophobic exposure or to form soluble off-pathway oligomers according to the recent findings [44], which showed that the ANS molecule may have a high binding affinity to the aggregates but not the surface of the MG. The accumulation of the folding intermediate usually leads to off-pathway aggregation, and CK can also form non-native oligomers when denatured in GdnHCl at high concentrations of 2 mg/ml [23] or 7.5 mg/ml [45]. However, no aggregation was detected during the equilibrium unfolding and refolding of all the five proteins under our protein concentration conditions (data not shown, see also reference [46]).

**Figure 5:**
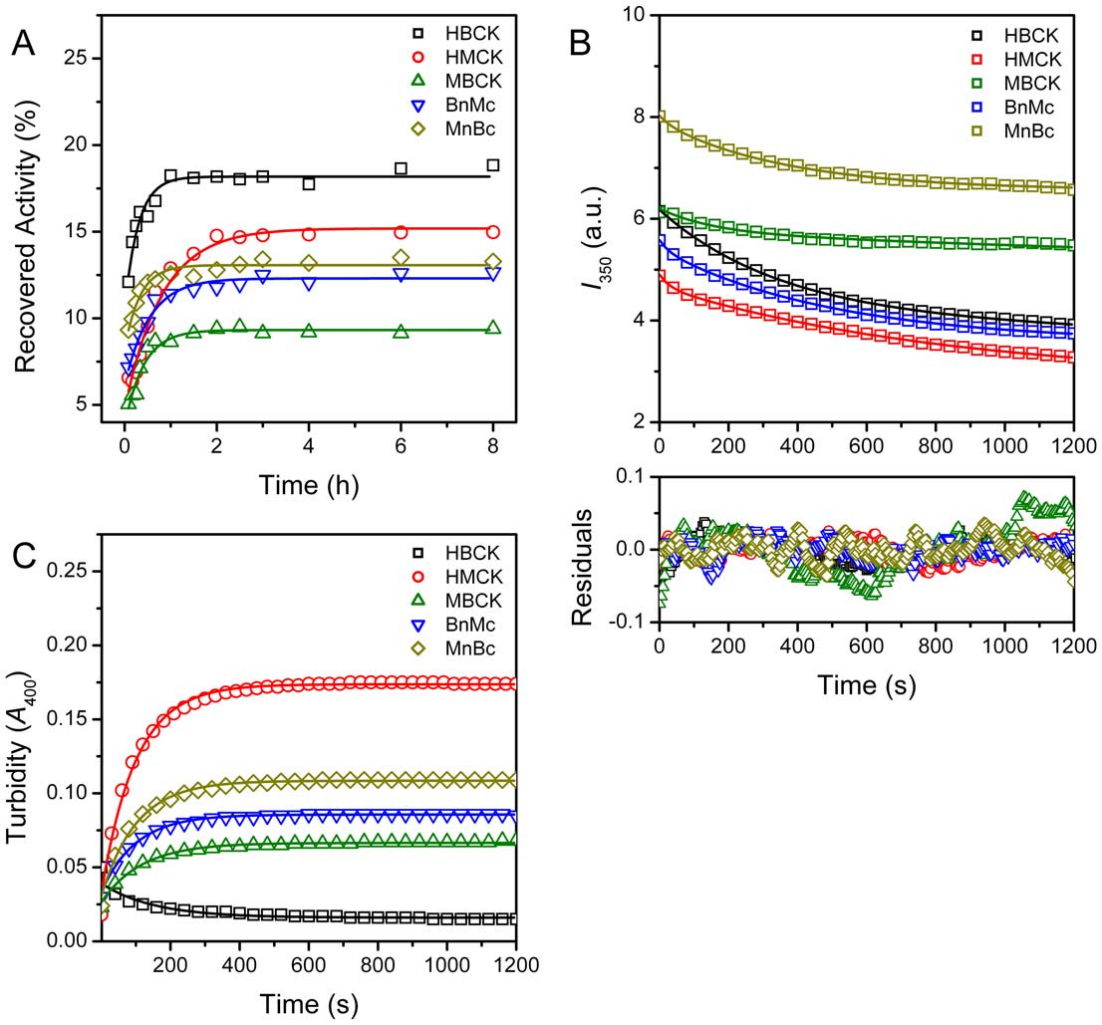
Reactivation, refolding and aggregation kinetics of CKs. (A) Refolding kinetics of CKs monitored by the intrinsic fluorescence emission at 350 nm (excited at 295 nm) versus time. (B) Reactivation kinetics monitored by the recovered activity of the samples quenched at suitable time intervals. The activity data were normalized by taking the activity of the native enzymes as 100%. (C) The time-course aggregation during refolding was monitored by the turbidity at 400 nm, and the data were recorded every 2 s. The refolding was initiated by manually diluting the fully denatured proteins in the refolding buffer with a mixing ratio of 1∶200 for refolding and reactivation and 1∶120 for aggregation. The reactivation and aggregation data were fitted by a single exponential kinetics, and the refolding data were fitted by the biphasic kinetics. The fitted curves are presented as solid lines.

**Figure 6:**
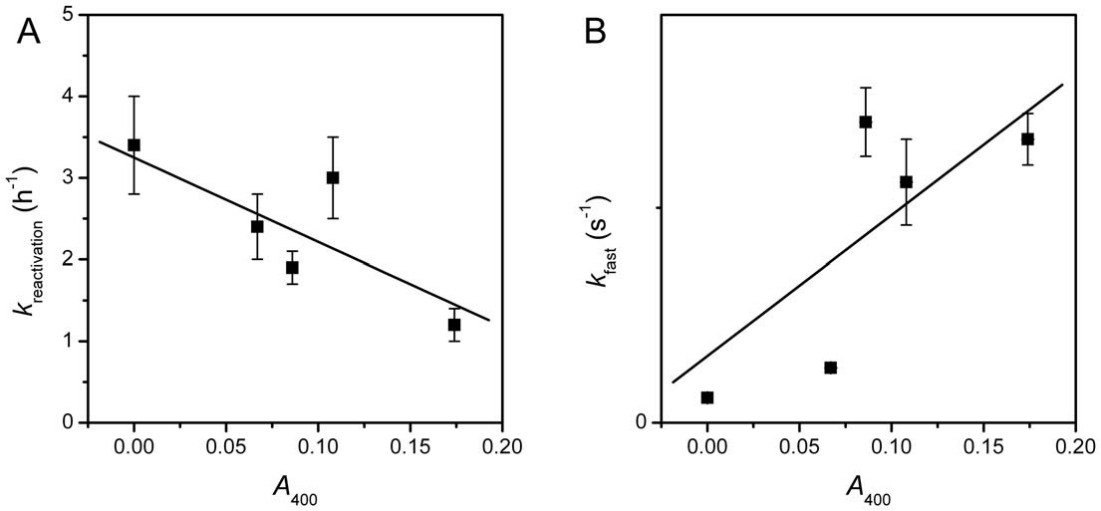
Correlation of the extent of aggregation (*A*_400_) with the refolding and reactivation rate constants. (A) *A*_400_ versus the reactivation rate constant. (B) *A*_400_ versus the fast-phase rate constant of refolding.

Previous studies have shown that the mutations impaired the domain interactions also affect the kinetic refolding and reactivation of RMCK [35], [42], [43]. As shown in Figure 5, the five proteins had different reactivation yields ranging from 9% to 18% under our conditions after dilution in the refolding buffer by fast manual mixing. HBCK had the highest reactivation yield, while MBCK was the lowest. The reactivation of HBCK was the fastest, while that of HMCK was the slowest (Table 3). The refolding of all five proteins followed a biphasic process when monitored by the fluorescence intensity change at 350 nm. The fast and slow phase rate constants of HMCK refolding were at the same level as those of RMCK [24], [35], [42], [43]. A comparison of the rate constants listed in Table 3 indicated that HMCK and the two chimeras had similar rate constants for both phases, which is about 10-fold faster than those of HBCK and MBCK. All proteins except HBCK aggregated seriously during kinetic refolding at a relatively higher protein concentration. HMCK had the most serious aggregation, followed by MnBc, BnMc and MBCK in a decreasing order. It seems that HBCK showed a small amount of aggregates in the initial stage of mixing, while the turbidity decreased continuously in a monophasic manner. This phenomenon was repeatable (data not shown). A possible explanation is that the fast manual mixing resulted in high local protein concentration. Nonetheless, the formation of aggregates of HBCK, if truly existed, seems to be reversible during the kinetic refolding process. The aggregation rates of the five proteins were similar, and the major difference was the extent of aggregation. A close inspection of the data in Table 3 indicated that the extent of aggregation is roughly inversely correlate to the rate constant of reactivation and positively correlate to the fast phase rate constant of refolding (Figure 6). However, no clear relationship was found among the reactivation yield, the extent of aggregation and the rate constants of refolding.

**Table 3:** Kinetic parameters of CK refolding, reactivation and aggregation during refolding.

	HBCK	HMCK	MBCK	BnMc	MnBc
*A*_400_ (a.u.)	n.a.	0.174±0.001	0.067±0.001	0.086±0.001	0.108±0.001
*k*_aggregation_ (×10^3^ s^−1^)	n.a.	10.1±0.1	8.6±0.1	9.8±0.1	10.0±0.1
Recovered Activity (%)	18.2±0.2	15.2±0.4	9.3±0.2	12.3±0.1	13.1±0.1
*k*_reactivation_ (h^−1^)	3.4±0.6	1.2±0.2	2.4±0.4	1.9±0.2	3.0±0.5
*k*_fast_ (×10^3^ s^−1^)	2.91±0.02	33±3	6.4±0.3	35±4	28±5
*k*_slow_ (×10^3^ s^−1^)	0.13±0.01	1.17±0.02	0.17±0.02	2.00±0.02	2.68±0.04

## Discussion

The majority of proteins in the cell belong to multidomain/multisubunit proteins due to the advantages in achieving complex and cooperative functions as well as precise regulations. Although the problem of protein folding has been studied extensively by using single-domain proteins as the model in the past several decades, the folding of multidomain/multisubunit proteins remains elusive due to the complexity raised by the folding of the individual domains and the assemble of these domains [47]. CK has been used as a model for the folding of dimeric two-domain proteins during the past 30 years. Previous studies have shown that RMCK folds via a complex pathway involving several folding intermediates [23]–[32]. However under normal folding conditions, the MG state is the major on-pathway equilibrium folding intermediate and the association/dissociation of the subunits is not a rate limiting step, as shown in Schemes 1 and 2. Mutational analysis indicates that the modification of either intra-subunit domain interactions or subunit-subunit interface significantly affects RMCK folding [35], [42], [43], [48]–[50], revealing the importance of the intra- and inter-subunit interactions between NTD and CTD in CK stability and folding. Moreover, all CK isoenzymes are highly conserved in their sequence, structure and catalytic properties, but differ in their tissue specificity, stability and physiological functions. These prominent features make CK isoenzymes to be an ideal system to explore the key dominants in multidomain/multimeric protein folding.

Consistent with the results in previous studies [21], [38], HBCK, HMCK and MBCK had quite dissimilar biochemical and biophysical properties although their three-dimensional structures are almost superimposed [12], [13]. Particularly, HMCK had the most compact structure, while HBCK had the loosest with large hydrophobic exposure and solvent-accessible Trp fluorophores. A comparison of the parameters of the five enzymes listed in Table 1 suggested that the catalytic efficiency and hydrophobic exposure were mainly determined by CTD, while the microenvironments of the Trp fluorophores were affected by the nature of domain interactions since all chimeras had similar *E*_max_ or *I*_320_/*I*_365_ value to HMCK. Two proteins, MBCK and BnMc, which had the smallest CD signals, were the most unstable. However, no obvious correlation could be identified between the tertiary and quaternary structural features monitored by spectroscopy and the thermodynamic parameters during CK folding (Table 2). These results suggested that neither the nature of NTD nor that of CTD significantly affect CK stability. Previously, we have found that the thermostability of HBCK is much lower than that of HMCK [21], while the results herein showed no significant difference in the GdnHCl-induced inactivation and unfolding between these two homodimers. This might be due to the different nature of protein inactivation and denaturation induced by heat or GdnHCl. Actually, previous studies have shown that RMCK undergoes dissimilar unfolding pathway when denatured by heat or chemical denaturants [22]. A close inspection of the domain organizations shown in Figure 1 suggested that the nature of the inter-subunit NTD-CTD interactions might affect the percentages of regular secondary structure contents and equilibrium folding of CK. The results herein were different from the significant changes in equilibrium folding caused by mutational analysis in literature [35], [42], [43], [48]–[50], which might caused by the very slight structural difference induced by domain swapping and large modifications by mutations. Unfortunately, it is difficult to predict the difference in inter-subunit domain interactions among the various patterns from the present research since only the structures of homodimers are available till now. The details may be clarified when the three-dimensional structure of MBCK or the chimeras could be resolved.

A much more obvious correlation could be obtained between the structural features and the kinetic refolding parameters. Three proteins (HMCK, BnMc and MnBc) that possessed almost identical Trp fluorophores under native states had similar fast- and slow-phase kinetic refolding parameters, while HBCK and MBCK showed another pattern. These results suggested that the incorporation of either NTD or CTD of HMCK in HBCK could alter the microenvironments of Trp fluorophores in HBCK via domain interactions, and further modify the refolding behavior of HBCK to be close to that of HMCK. This deduction is consistent with the previous observation that Trp residues play a crucial role in CK stability and folding by mutational analysis [48]. Our results further suggested that the assembly of domains, which influenced the packing of residues around Trp fluorophores in CTD, played a crucial role in determining the folding rates of CK. Meanwhile, it seems that the folding rates correlated with the occurrence of aggregation. No serious aggregation was observed during the dilution-initiated refolding of HBCK, while the proteins contained at least one domain from the M type aggregated seriously. It is reasonable that for proteins with multiple kinetic intermediates, the folding rate determines the extent of the accumulation of aggregation-prone intermediate(s), and thus the faster an aggregation-prone intermediate accumulates, the more serious aggregation occurrs. As for the chimeras, the fast-phase rate constants were similar to HMCK, but the slow-phase rate constants were about 2-fold larger than that of HMCK. The fast slow-phase transition decreased the number of aggregation-prone intermediate and the extent of aggregation thereby. Furthermore, the lower extent the protein aggregated, the higher rate the enzyme reactivated.

It is interesting that one of the chimera MnBc had similar high catalytic efficiency to HBCK, slightly higher stability of the native protein than HMCK for the N to I transition, and very fast refolding and reactivation rate. In other words, the chimera MnBc had most of the advantages of the two natural isoenzymes except for a moderate extent of aggregation during refolding. Thus our study also provided a way for improving the properties of enzymes in biotechnology by modifying the domain interactions. Since isoenzymes are widely distributed in the cell, this strategy may also work for the other multidomain proteins. Further mutational analysis could determine the key residues involved in the dissimilar properties of the isoenzymes, and stability or activity improvements could thus screened based on the difference in isoenzyme sequences.
